# Multicenter evaluation of Verigene Enteric Pathogens Nucleic Acid Test for detection of gastrointestinal pathogens

**DOI:** 10.1038/s41598-021-82490-z

**Published:** 2021-02-04

**Authors:** Kosuke Kosai, Hiromichi Suzuki, Kiyoko Tamai, Yuya Okada, Norihiko Akamatsu, Atsuo Ueda, Shigeyuki Notake, Yuji Yaguchi, Katsunori Yanagihara

**Affiliations:** 1 Department of Laboratory Medicine, Nagasaki University Hospital, 1-7-1 Sakamoto, Nagasaki, Nagasaki 852-8501 Japan; 2 Division of Infectious Diseases, Department of Medicine, Tsukuba Medical Center Hospital, Ibaraki, Japan; 3 Miroku Medical Laboratory Inc., Nagano, Japan; 4 Department of Clinical Laboratory, Tsukuba Medical Center Hospital, Ibaraki, Japan; 5 Department of Laboratory Medicine, Nagasaki University Graduate School of Biomedical Sciences, Nagasaki, Japan

**Keywords:** Microbiology, Infectious diseases

## Abstract

We investigated the efficiency of the Verigene Enteric Pathogens Nucleic Acid Test (Verigene EP test), which is an automated microarray-based assay system that enables rapid and simultaneous genetic detection of gastrointestinal pathogens and toxins, including those in the *Campylobacter* Group, *Salmonella* species, *Shigella* species, the *Vibrio* Group, *Yersinia enterocolitica*, Shiga toxin 1 and 2, norovirus GI/GII, and rotavirus A. Three clinical laboratories evaluated the Verigene EP test, using 268 stool samples for bacterial and toxin genes and 167 samples for viral genes. Culture-based reference methods were used for the detection of bacteria and toxins, while a different molecular assay was used for viral detection. The overall concordance rate between the Verigene EP test and the reference methods for the 1940 assays was 99.0%. The overall sensitivity and specificity of the Verigene EP test were 97.0% and 99.3%, respectively. Of the 19 samples with discordant results, 13 samples were false positives and six were false negatives. The Verigene EP test simultaneously detected two targets in 11 samples; overall, the test demonstrated high efficiency in detecting crucial diarrheagenic pathogens, indicating its suitability for clinical practice.

## Introduction

Acute infectious diarrhea is a major cause of outpatient visits and hospitalization^1^. Since most diarrheal illnesses are self-limited, microbiological testing is not necessary for all patients. However, rapid etiological identification is required for those with serious symptoms and conditions such as systemic illness, fever, and bloody stool^2^, to enable effective antimicrobial treatment against specific pathogens. Furthermore, because some patients with infectious diarrhea should be isolated to prevent pathogen transmission in hospitals, more prompt and accurate methods are needed to detect crucial gastrointestinal pathogens.

The Verigene Enteric Pathogens Nucleic Acid Test (Verigene EP test) (Luminex Corporation) is an automated system based on microarray technology that enables simultaneous detection of enteric pathogens faster (3 h or less, including handling time) than conventional stool culture^3^. Since only a few previous studies have investigated the utility of this test^3,4^, and epidemiological differences affect the performance of diagnostic methods^5^, we conducted a multicenter evaluation in Japan to assess the ability of the Verigene EP test to detect crucial enteric pathogens.

## Materials and methods

### Study design

We examined clinical stool samples submitted to laboratories at the Nagasaki University Hospital, Tsukuba Medical Center Hospital, and Miroku Medical Laboratory between October 2016 and March 2018. The assay for this study was performed soon after routine testing or using samples stored at − 80 °C.

All research was performed in accordance with the Declaration of Helsinki and the Ethical Guidelines for Medical and Health Research Involving Human Subjects, which was issued by the Japanese Ministry of Education, Culture, Sports, Science and Technology and the Ministry of Health, Labour and Welfare. Informed consent was obtained using the opt-out method provided on the website. The Institutional Review Board of Nagasaki University Hospital approved this study (approval number 16062725/18070902).

### Verigene EP test

The Verigene EP test, which has been approved for use in clinical settings by the Food and Drug Administration (FDA) in the United States and by the Pharmaceuticals and Medical Devices Agency (PMDA) in Japan, was used according to the manufacturer’s instructions. The test requires a processor, a reader, and single-use consumables. To prepare the fecal suspension for the assay, a swab dipped into stool was mixed with Stool Prep Buffer for 15–20 s using a vortex mixer. Extraction Tray, Tip Holder Assembly, Amplification Tray, and Test Cartridge were loaded into the processor. After centrifugation, 200 µL of the suspension was loaded into the Sample Loading Well of the Extraction Tray. Nucleic acid extraction, amplification, and nanoparticle-based microarray hybridization were automatically performed on the processor, and the data were analyzed by the reader. The Verigene EP test is aimed at the genetic detection of *Campylobacter* Group (*C. jejuni*, *coli*, and *lari*), *Salmonella* species, *Shigella* species (*S. boydii*, *sonnei*, *flexneri*, and *dysenteriae*), *Vibrio* Group (*V. cholerae* and *parahaemolyticus*), *Yersinia enterocolitica*, Shiga toxin (Stx) 1 and 2, norovirus GI/GII, and rotavirus A^3,4,6^. The test does not target protozoa.

### Culture methods

Culture-based methods for the detection of bacteria and toxins were performed based on the guidelines for the diagnostic testing of enteric infections, textbooks, and the manufacturer’s instructions^7–9^. The media used and the identification methods are listed in Supplemental Table 1. We did not use enrichment broth for the cultures. In addition to cultures, reversed passive latex agglutination, and enzyme immunoassay were used for detecting Shiga toxins.

### xTAG Gastrointestinal Pathogen Panel

The assay was performed with the FDA-approved xTAG Gastrointestinal Pathogen Panel (GPP) (Luminex Corporation), according to the manufacturer’s instructions. xTAG GPP requires prior nucleic acid extraction, multiplex amplification, and bead hybridization; the test can detect the genes of 15 gastrointestinal pathogens and toxins (nine bacteria and toxins, three viruses, and three protozoa), with several recent studies validating its performance^3,10–12^. An inoculating loop dipped into stool or 100 μL of watery diarrheal stool was added to a Bertin SK38 Soil Grinding Lysis Bead Tube with NucliSENS easyMAG Lysis Buffer (bioMérieux) or ASL Lysis Buffer (Qiagen). Ten μL of xTAG MS2 (internal control) was added, except in the negative control tube. The tube was mixed for 5 min using a vortex mixer, settled at room temperature for 10–15 min, and centrifuged at 14,000 rpm for 2 min. 200 μL of the supernatant was removed from the middle of the layer and used for nucleic acid extraction, using the QIAamp MinElute Virus Spin Kit (Qiagen) according to the manufacturer’s instructions. Ten μL of the nucleic acid sample was mixed with 15 μL of prepared multiplex PCR master mix, and the PCR reactions were performed under the following conditions: 20 min at 53 °C, 15 min at 95 °C, 38 cycles consisting of 30 s at 95 °C, 30 s at 58 °C, 30 s at 72 °C, and 2 min at 72 °C for the final extension. Five μL of the PCR product was mixed with 20 μL xTAG GPP Bead Mix and 75 μL xTAG SAPE (Streptavidin, R-Phycoerythrin Conjugate) diluted 75 times with xTAG Reporter Buffer. Bead hybridization was performed under the following conditions: 3 min at 60 °C and 45 min at 45 °C. Data were analyzed using the xTAG Data Analysis Software LSM version 2.00 after data acquisition using the MAGPIX System^13^.

### Multiplex real-time PCR

Multiplex real-time PCR assay for viral detection was performed by BML, INC. as follows^14,15^. Briefly, a 10% (wt/vol) stool suspension was prepared with distilled sterile water and centrifuged at 3000×*g* for 20 min. Nucleic acid was extracted from 140 μL of the stool suspension using NucleoMag (Takara Bio Inc.) or QIAamp Virus RNA Mini Kit (Qiagen) according to the manufacturer’s instructions. The extracted nucleic acid was eluted with 60 μL of diethyl pyrocarbonate-treated water and stored at – 80 °C until use. After reverse transcription using PrimeScript 1st strand cDNA Synthesis Kit (Takara Bio Inc.) according to the manufacturer’s instructions, multiplex PCR was performed using the QuantiTect Multiplex PCR kit (Qiagen) on a LightCycler 480 System (Roche Diagnostics). Amplification was carried out in 50 μL reaction mixture containing 5 μL nucleic acid sample. The reaction steps were as follows: 15 min at 95 °C and 40 cycles consisting of 1 min at 94 °C and 90 s at 60 °C. Primers and probes for the detection of norovirus GI/GII and rotavirus A genes have been described elsewhere^14,15^.

### Reference methods and discrepant analysis

Culture-based methods were used as reference methods for the detection of bacteria and toxins. The xTAG GPP was used as a reference method for viral detection. For the detection of bacteria and toxins, a discrepant analysis was performed by re-examination using the Verigene EP test for the samples with false-negative results or by the assay using the xTAG GPP for the samples with false-positive results. Discrepant analysis for each virus was performed using multiplex real-time PCR.

### Efficiency of the Verigene EP test

We calculated the sensitivity and specificity of the Verigene EP test in detecting targets, based on results from the reference methods, and determined the 95% confidence intervals using the software R version 3.5.1^16^.

### Assessment using characterized stool samples

To evaluate the ability of the Verigene EP test to detect pathogens present only in a few clinical samples, we assessed characterized stool samples. A stool sample obtained from BizCom Japan, Inc., which was confirmed to be negative for the target pathogens by the Verigene EP test and culture, was used for this analysis. To prepare 200 μL of characterized samples, 8 μL of bacterial suspension, 160 μL of the Stool Prep Buffer, and 32 μL of stool were mixed. The target bacterial concentrations in the characterized samples were twice or 20 times the limit of detection (LoD) of the Verigene EP test. The LoD information was provided by the Luminex Corporation, and the LoD used in this study was 1.11 × 10^5^ colony-forming units (CFU)/mL, which was the highest LoD among *Shigella* species. The concentration of the bacterial suspension was adjusted using the McFarland turbidity standard. Serial dilutions of bacterial suspensions were plated onto agar medium, and the actual concentration was calculated by counting the number of bacterial colonies.

## Results

### Detection ability of Verigene EP test for clinical stool samples

We examined 268 clinical stool samples for bacteria and toxins (266 samples for Stx 1 and 2) and 167 samples for viruses. Of the 268 samples tested for bacteria and toxins and the 167 samples tested for viruses, 256 and 160 samples respectively (95.5% and 95.8%) had fully concordant results between the Verigene EP test and the reference methods. Table 1 depicts the detection ability of the Verigene EP test for clinical samples as compared to the reference methods. The overall sensitivity and specificity of the test were 97.0% (195/201) and 99.3% (1726/1739), respectively. The sensitivity ranged from 87.5% for rotavirus (7/8) to 100.0% for the *Campylobacter* Group (47/47), *Shigella* species (2/2), *Y. enterocolitica* (16/16), and Stx 1 and 2 (25/25). Similarly, the specificity ranged from 97.7% for norovirus GI/GII (126/129) to 100.0% for *Shigella* species (266/266) and the *Vibrio* Group (259/259).

**Table 1:** Efficiency of the Verigene Enteric Pathogens Nucleic Acid Test to detect pathogens and toxin genes for clinical stool samples.

Pathogen and toxin gene	TP	TN	FP	FN	Sensitivity (%)	Specificity (%)
**Bacterium**						
*Campylobacter* Group^a^	47	219	2^d^	0	100.0 (92.5–100.0)	99.1 (96.8–99.9)
*Salmonella* species	53	211	1^e^	3^f^	94.6 (85.1–98.9)	99.5 (97.4–100.0)
*Shigella* species^b^	2	266	0	0	100.0 (15.8–100.0)	100.0 (98.6–100.0)
*Vibrio *Group^c^	8	259	0	1^f^	88.9 (51.8–99.7)	100.0 (98.6–100.0)
*Yersinia enterocolitica*	16	249	3^g^	0	100.0 (79.4–100.0)	98.8 (96.6–99.8)
Shiga toxin 1 and 2	25	239	2^h^	0	100.0 (86.3–100.0)	99.2 (97.0–99.9)
**Virus**						
Norovirus GI/GII	37	126	3^d^	1^e^	97.4 (86.2–99.9)	97.7 (93.4–99.5)
Rotavirus A	7	157	2^h^	1^d^	87.5 (47.3–99.7)	98.7 (95.5–99.8)
Overall	195	1726	13	6	97.0 (93.6–98.9)	99.3 (98.7–99.6)

Data were compared to reference methods and expressed as numbers or percentages (95% confidence interval).

*TP* true positive, *TN* true negative, *FP* false positive, *FN* false negative.

^a^*Campylobacter jejuni*, *coli*, and *lari.*

^b^*Shigella boydii*, *sonnei*, *flexneri*, and *dysenteriae.*

^c^*Vibrio cholerae* and *parahaemolyticus.*

^d^All samples were positive in the discrepant analysis.

^e^The sample was negative in the discrepant analysis.

^f^All samples were positive in re-examination using the Verigene EP test.

^g^One sample was positive and two samples were negative in the discrepant analysis.

^h^One sample was positive and the other was negative in the discrepant analysis.

Of the 19 samples with discordant results, 13 and six were false positives and false negatives, respectively. Seven of the 13 false-positive samples were positive, and one out of the six false-negative samples was negative in the discrepant analysis (Table 1).

The Verigene EP test simultaneously detected two targets in 11 samples (Table 2). Of these 11 samples, five samples showed fully concordant results between the Verigene EP test and the reference methods, whereas six samples exhibited partial concordance between the two (Table 2).

**Table 2:** Detection of multiple targets by the Verigene Enteric Pathogens Nucleic Acid Test and comparative analysis.

Detection by Verigene EP test	Detection by reference methods	Results of discrepant analysis
*Campylobacter *Group and *Salmonella* species	*Campylobacter *Group and *Salmonella* species	–
*Campylobacter *Group and *Vibrio* Group	*Campylobacter *Group and *Vibrio* Group	–
*Campylobacter* Group and rotavirus A	*Campylobacter* Group only	Negative for rotavirus A
*Salmonella* species and *Y. enterocolitica*	*Salmonella* species only	Positive for *Y. enterocolitica*
*Salmonella* species and norovirus GI/GII	*Salmonella* species and norovirus GI/GII	–
*Vibrio* Group and *Y. enterocolitica*	*Vibrio* Group only	Negative for *Y. enterocolitica*
*Vibrio* Group and Shiga toxin	*Vibrio* Group only	Positive for Shiga toxin
*Y. enterocolitica* and Shiga toxin	*Y. enterocolitica* only	Negative for Shiga toxin
Shiga toxin and norovirus	Shiga toxin and norovirus	–
*Y. enterocolitica* and norovirus GI/GII	*Y. enterocolitica* and norovirus GI/GII	–
*Y. enterocolitica* and norovirus GI/GII	Norovirus GI/GII only	Negative for *Y. enterocolitica*

### Detection ability of Verigene EP test for characterized stool samples

Since the clinical stool samples included only two samples positive for *Shigella* species, we assessed the ability of Verigene EP test to detect the pathogen using characterized stool samples. We used 26 *Shigella sonnei* strains, which were previously isolated and stored in our institutions, and 13 strains were used in the preparation of characterized stool samples with concentrations targeting twice or 20 times the LoD of the Verigene EP test. The actual bacterial concentrations of these samples were 0.1–2.6 × 10^5^ CFU/mL or 5.0 × 10^5^–1.0 × 10^6^ CFU/mL. Of the 26 characterized samples examined, the Verigene EP test correctly detected *S. sonnei* in 25 samples, except for one sample with a bacterial concentration of 2.6 × 10^5^ CFU/mL.

## Discussion

Our study results demonstrated that the Verigene EP test accurately detected the genes of enteric pathogens and toxins in clinical samples with high sensitivity and specificity, which is consistent with previous reports^3,4^. Due to the ability of the test to detect multiple targets in a single assay, the Verigene EP test can avoid overlooking of any crucial gastrointestinal pathogens. Furthermore, the Verigene EP test enables rapid diagnosis with less work or training for laboratory technicians^12^; on the other hand, conventional bacterial cultures are labor- and time-intensive. Additionally, unnecessary use of antimicrobial drugs and the duration of patient isolation may be reduced because of rapid diagnosis. A previous study reported that the xTAG GPP for gastrointestinal pathogens increased laboratory testing costs but decreased total costs for hospitalization by reducing patient isolation days^3,17^. The Verigene EP test might have similar effects; however, further studies are required.

While the Verigene EP test showed 19 discrepant results compared to the reference methods, the discrepant analysis proved that the Verigene EP test might have provided true results, at least in some samples with discrepant results. If bacteria were detected by molecular method but not cultured, we should recognize both possibilities; that the results of molecular methods may be false positives and that the sensitivity of cultures is lower than that of molecular methods^18–20^. A previous study described that the culture method failed to detect *Campylobacter* in 30% of positive clinical samples compared to non-cultural methods, including molecular assays^21^. Additionally, if the xTAG GPP was not the best test in some cases, as described previously^11^, the false-positive results of the Verigene EP test could result from the false-negative results of the xTAG GPP in the discrepant analysis.

Although each institution needs to consider how best to use the Verigene EP test effectively in daily practice, the test may be suitably used for community-acquired diarrhea requiring hospitalization to rapidly screen unmissable pathogens. Conversely, for hospital-acquired diarrhea, we might have to initially perform the Verigene *Clostridium difficile* nucleic acid test, which is a separate test for a single pathogen and performed using the same Verigene System^12,22^.

This study has some limitations. First, we could not adequately assess the ability of this test to detect *Shigella* species in clinical samples because the number of positive samples was limited. Instead, the Verigene EP test could successfully detect *S. sonnei* in characterized stool samples, and previous studies reported that the sensitivity and specificity of the Verigene EP test for the detection of *Shigella* species was 87.5–95.4% and 99.1–99.8%, respectively^3,4^. Furthermore, because we did not collect patient information and did not test all samples submitted to our laboratories during the study period, we could not validate the pathogenicity of the detected pathogens and the incidence of infections. Reportedly, some Stx 1 and 2 subtypes are linked to mild diseases or are rarely involved in human diseases^23^. Finally, for detecting bacteria and toxins, we could not assess the efficiency of the Verigene EP test compared to other molecular assays because another molecular method was used only for the discrepant analysis.

In conclusion, the Verigene EP test is useful for detecting crucial diarrheagenic pathogens, and may have applications in daily clinical practice. Further studies are needed to establish practical diagnostic strategies for the complementary use of molecular assays and traditional methods.

## Supplementary Information

Supplementary Information.
