# Supplementary material for: Multicenter evaluation of Verigene Enteric Pathogens Nucleic Acid Test for detection of gastrointestinal pathogens
Source: Sci Rep. 2021 Feb 4;11:3033. doi: 10.1038/s41598-021-82490-z (PMC7862589; doi:10.1038/s41598-021-82490-z)
Supplement: Supplementary file 1 — Supplementary Information. [file 41598_2021_82490_MOESM1_ESM.doc]

**Title**

**Multicenter evaluation of Verigene Enteric Pathogens Nucleic Acid Test for detection of gastrointestinal pathogens**

**Authors**

Kosuke Kosai^a^,*****, Hiromichi Suzuki^b^, Kiyoko Tamai^c^, Yuya Okada^a^, Norihiko Akamatsu^a^, Atsuo Ueda^d^, Shigeyuki Notake^d^, Yuji Yaguchi^c^, Katsunori Yanagihara^a,e^

**Affiliations**

^a^Department of Laboratory Medicine, Nagasaki University Hospital, Nagasaki, Japan

^b^Division of Infectious Diseases, Department of Medicine, Tsukuba Medical Center Hospital, Ibaraki, Japan

^c^Miroku Medical Laboratory Inc., Nagano, Japan

^d^Department of Clinical Laboratory, Tsukuba Medical Center Hospital, Ibaraki, Japan

^e^Department of Laboratory Medicine, Nagasaki University Graduate School of Biomedical Sciences, Nagasaki, Japan

***Correspondence**

Kosuke Kosai, MD, PhD

Department of Laboratory Medicine, Nagasaki University Hospital

1-7-1 Sakamoto, Nagasaki, Nagasaki 852-8501, Japan

Tel: +81-95-819-7574; Fax: +81-95-819-7422

E-mail: [k-kosai@nagasaki-u.ac.jp](mailto:k-kosai@nagasaki-u.ac.jp)

Supplemental Table 1. Reference methods used for detection of bacteria and toxins in this study.

| Bacteria and toxins | Medium | Identification |
| --- | --- | --- |
| *Campylobacter* Group  *Salmonella* species  *Shigella* species  *Vibrio* Group  *Yersinia enterocolitica*  Shiga toxin 1 and 2 | POURMEDIA MODIFIED SKIRROW AGAR (EIKEN CHEMICAL CO., LTD.)  PLATE MOD CAMPYLOBACTER AGAR 10% SB (Nippon Becton Dickinson Company, Ltd.)  Nissui Plate Skirrow Agar, Modified (NISSUI PHARMACEUTICAL CO., LTD.)  CHROMagar STEC/SS extra (KANTO CHEMICAL CO., INC.)  PLATE MOD SALMONELLA SHIGELLA (Nippon Becton Dickinson Company, Ltd.)  SS agar (SS) (bioMérieux Japan Ltd.)  CHROMagar STEC/SS extra (KANTO CHEMICAL CO., INC.)  PLATE MOD SALMONELLA SHIGELLA (Nippon Becton Dickinson Company, Ltd.)  SS agar (SS) (bioMérieux Japan Ltd.)  KYOKUTO VITAL MEDIA TCBS Agar (KYOKUTO PHARMACEUTICAL INDUSTRIAL CO., LTD.)  PLATE TCBS AGAR (Nippon Becton Dickinson Company, Ltd.)  PLATE CIN AGAR (Nippon Becton Dickinson Company, Ltd.)  CHROMagar STEC/SS extra (KANTO CHEMICAL CO., INC.)  CHROMagar STEC (KANTO CHEMICAL CO., INC.)  "KBM"ST-SS/MC-MAC II Agar (Kohjin Bio Co., Ltd.) | Api Campy (bioMérieux Japan Ltd.)  MALDI Biotyper (Bruker Daltonik GmbH)  Neg EN Combo 1J (Beckman Coulter, Inc.)  MALDI Biotyper (Bruker Daltonik GmbH)  Salmonella as set 1 "SEIKEN" (DENKA SEIKEN CO., LTD.)  Shigella as set 3 "SEIKEN" (DENKA SEIKEN CO., LTD.)  Neg EN Combo 1J (Beckman Coulter, Inc.)  Neg EN Combo 1J (Beckman Coulter, Inc.)  MALDI Biotyper (Bruker Daltonik GmbH)  Neg EN Combo 1J (Beckman Coulter, Inc.)  MALDI Biotyper (Bruker Daltonik GmbH)  VTEC-RPLA "SEIKEN" (DENKA SEIKEN CO., LTD.)  PREMIER EHEC (Meridian Bioscience, Inc.) |

Each laboratory used one of the media and at least one identification method among those listed.
